# Reports on Hospitals of the United Kingdom

**Published:** 1913-11-08

**Authors:** Henry Burdett


### REPORTS ON
### Hospitals of the United Kingdom.

By SIR HENRY BURDETT, K.C.B., K.C.V.O.

SERIES III.

In resuming these reports, we desire to say how much we have appreciated the numerous spontaneous letters which have reached us from many hospitals in various parts of the United Kingdom, requesting that they may be inspected and included in these reports. These requests have placed us in a great difficulty, because a large proportion of them have come from institutions which are situated in districts which we have already visited, and it is impossible to visit them again, at any rate until the whole of our inspections are completed, which should be, in the ordinary course, by the end of the present year. We hope, therefore, that the managers of every hospital or kindred institution of which an inspection is desired will kindly understand that we will bear every such request in mind, and that if and when it may be possible we will certainly not fail to pay them a visit as they wish. Out of the large number of requests so received we have taken all which relate to institutions situated within the counties embraced by our present autumn tour. We hope we may add that we feel it a great compliment to have these requests, which are indeed sincerely welcomed, for these inspections have entailed grave responsibilities.

### CHELMSFORD AND ESSEX HOSPITAL, CHELMSFORD.

This institution was established as a dispensary in 1818; it commenced work as a hospital in 1871, and was enlarged and brought up to its present state of structural efficiency in 1908-9. Commenced first at a house in Duke Street, near the railway station, inadequate accommodation led to several removals, and ultimately the present site was purchased and the initial hospital and dispensary buildings were started in 1882. In 1908 a public meeting, organised by Mr. C. E. Ridley, the active and successful Chairman of the Building Committee, desired to enlarge the original building and raised £7,000 to defray the entire cost. The institution owes much to the successful advocacy of Mr. C. E. Ridley and to his devoted personal services. We have no doubt that he will use his influence to put right the various matters to which we venture to call attention in the following report.

### The Condition of the Hospital.

We inspected this hospital on Saturday, October 18, 1913, arriving about 3 p.m. The matron was absent, it being her off-day, we were informed by the sister of the ground floor and casualty department, who kindly accompanied us through the hospital. It is but just to say that the meeting held in 1908 which secured the £7,000 then raised has been wisely expended and has produced some excellent buildings, which leave little to desire in so small a hospital as one of forty-three beds. This is a bright, airy and attractive-looking hospital inside and out. The beds and other fittings, with the exception of the larger combined tables in each ward, are on the whole good. The design and proportions of the combined ward tables have not been carefully thought out, with the result that they contain heavy drawers which are clumsy from their length. It would be well to have these drawers divided into two. It is true that splints are at present kept in these drawers, but it would be easier to keep them all together in a splint room, where they would be in constant readiness for use in any part of the hospital.

We noticed that flock is used in some portions of the hospital, and wherever it is so used horse-hair should be quickly substituted. Everything in the large ward upstairs was in excellent order, and we regret that the sister should have been off-duty at the time of our visit. She was, we understand, trained at Kettering, and if so the authorities at that hospital may have some pride in her work. Her drawers, wards, cupboards, and every detail were in excellent order with the exception of the bath, which is apparently not in much request on this floor, but had recently been used for flowers. It was not so smart as it might have been. We found the theatre in good order, but the lavatory fittings are not satisfactory. They are calculated to retain septic matter, and they cannot be thoroughly cleaned in every part. This defect condemns them as improper for hospital use. The casualty department was exceedingly well kept and in perfect order throughout. The lavatories and some other portions of the ward units in the hospital presented an untidy appearance, and seem to require much closer supervision, and the enforcement of a better system in regard to details than appears to prevail at present.

We gained the impression that the whole place, regarded administratively, might be smartened up, for such a step would much improve the general atmosphere and the working. We noticed, for instance, that in the children's ward, though the children were having their tea, there was no probationer or other attendant present, which surprised us.

### Paying Patients.

There are two small wards for the reception of paying patients, who can be attended by their private doctors, the price charged for hospital care being from two guineas a week each patient, exclusive of medical charges. We imagine this to be a comfortable hospital for patients, but it would be improved by an increase in the quality of its administrative supervision.

Nursing and Domestic Accommodation.

The nursing and domestic accommodation was good, but in one of the maids' rooms, containing four beds, there were only two wash-basins, although four are required. We noticed, too, that the nurses' sitting-room is not as comfortable for off-duty purposes as it could and ought to be made. Three restful chairs are not enough for the comfort of ten nurses when off duty.

The Secretary.

The Secretary of this hospital is Miss Haigh, who also fulfils the duties of collector, dispenser, and developer of the Röntgen ray and other photographs. She must have her hands full, we imagine, and it was matter for regret that we did not have an opportunity of making her acquaintance and learning the nature and extent of her duties.

An Experience of National Insurance.

The report for the year 1912 is an interesting document, and courageously grasps the difficulties that have arisen and are likely to arise in consequence of the introduction of national insurance. The experience of the Chelmsford Hospital appears to be that the Act is likely to lead to some loss of income and to bring to the hospital more in-patients than heretofore, who will require special medical and surgical skill for which the Act makes no provision. A comparison for the last three years seems to show that the in-patients, number of operations, casualties, and dental cases are increasing on the one hand, whilst the number of dispensary patients and attendances are decreasing. Of the forty-three beds the average number occupied was 32.6 in 1912, and the average number of days each patient was resident was 19.2. Chelmsford has a Hospital Working Party which contributed £125 in January 1912. There is a sum set aside as a Convalescent Fund, which rendered material aid to several patients at convalescent homes. We are glad to note that efforts are being made with success to interest the working classes in the support of this institution. The report contains a suggestive statement by the House Committee, formulating a set of regulations to cover the changes arising out of the National Insurance Act. These regulations make no alteration in the old system for the treatment of accidents and emergencies, but the Committee look for the co-operation of the Insurance Commissioners and approved societies to make all the special payments they have the power to provide. Out-patients who are insured are to be treated once and then referred to their panel doctor, except where, in the opinion of the honorary medical staff, a particular case cannot receive proper treatment at home. The treatment of insured patients in the Dispensary Department will be given up, and a form of questions has been prepared, which all in-patients (insured or uninsured) or their responsible representatives will be required to answer and sign. To enable the Committee to fix the weekly payment each in-patient will be required to make to the hospital a return enabling them to fix the rate in each case, and a minimum sum of 3s. per patient has, heretofore, been enforced. The hospital deserves the support of the community which it serves, and the latter may reasonably be called upon to increase their contributions by £500 a year, and so to put the institution upon a sound financial basis.

